# The Effects of Intermittent Fasting and Continuous Energy Restriction with Exercise on Cardiometabolic Biomarkers, Dietary Compliance, and Perceived Hunger and Mood: Secondary Outcomes of a Randomised, Controlled Trial

**DOI:** 10.3390/nu14153071

**Published:** 2022-07-26

**Authors:** Stephen Keenan, Matthew B. Cooke, Won Sun Chen, Sam Wu, Regina Belski

**Affiliations:** 1 School of Health Sciences, Swinburne University of Technology, Hawthorn, VIC 3122, Australia; mbcooke@swin.edu.au (M.B.C.); wchen@swin.edu.au (W.S.C.); sswu@swin.edu.au (S.W.); regina.belski@latrobe.edu.au (R.B.); 2 School of Allied Health, Human Services and Sport, La Trobe University, Bundoora, VIC 3086, Australia

**Keywords:** intermittent fasting, continuous energy restriction, compliance, cardiometabolic

## Abstract

(1) Background: Excess weight in the form of adiposity plays a key role in the pathogenesis of cardiometabolic diseases. Lifestyle modifications that incorporate continuous energy restriction (CER) are effective at inducing weight loss and reductions in adiposity; however, prescribing daily CER results in poor long-term adherence. Over the past decade, intermittent fasting (IF) has emerged as a promising alternative to CER that may promote increased compliance and/or improvements in cardiometabolic health parameters independent of weight loss. (2) Methods: This paper presents a secondary analysis of data from a 12-week intervention investigating the effects of a twice-weekly fast (5:2 IF; IFT group) and CER (CERT group) when combined with resistance exercise in 34 healthy participants (17 males and 17 females, mean BMI: 27.0 kg/m^2^, mean age: 23.9 years). Specifically, changes in cardiometabolic blood markers and ratings of hunger, mood, energy and compliance within and between groups were analysed. Dietary prescriptions were hypoenergetic and matched for energy and protein intake. (3) Results: Both dietary groups experienced reductions in total cholesterol (TC; mean reduction, 7.8%; *p* < 0.001), low-density lipoprotein cholesterol (LDL-C; mean reduction, 11.1%; *p* < 0.001) and high-density lipoprotein cholesterol (mean reduction 2.6%, *p* = 0.049) over the 12 weeks. Reductions in TC and LDL-C were greater in the IFT group after adjustment for baseline levels and change in weight. No significant changes in markers of glucose regulation were observed. Both groups maintained high levels of dietary compliance (~80%) and reported low levels of hunger over the course of the intervention period. (4) Conclusions: Secondary data analysis revealed that when combined with resistance training, both dietary patterns improved blood lipids, with greater reductions observed in the IFT group. High levels of compliance and low reported levels of hunger throughout the intervention period suggest both diets are well tolerated in the short-to-medium term.

## 1. Introduction

Overweight and obesity, conditions generally accompanied by excessive body fat, are a common and growing health concern worldwide [1]. Excess adiposity is associated with insulin resistance [2], derangement of blood lipids [3] and systemic inflammation [4], which can increase an individual’s risk of developing cardiovascular disease [5], insulin resistance [6] and type 2 diabetes [7]. Whereas excess adiposity is a common phenotypic characteristic of overweight and obesity, individuals that fall within the normal-weight body mass index (BMI) category can also display high body fat percentage. These individuals, typically referred to as metabolically obese, normal-weight (MONW), are often characterized by excess visceral adipose tissue and ectopic fat deposition and show some degree of metabolic dysregulation [8]. Given the deleterious effects of excess adiposity on disease risk, it is clear that regardless of BMI, reducing weight, especially in the form of adipose tissue, could be an important strategy for improving the health of a large proportion of the population.

Individuals can utilise various strategies to lose weight. Lifestyle interventions that promote reduced energy intake and increased physically activity are amongst those most commonly prescribed by health and clinical practitioners. Whereas continuous energy restriction (CER) has been the more traditional method to decrease energy intake, intermittent fasting (IF) has emerged over the past 10 years as a potential alternative [9]. Unlike CER, which consists of a daily energy restriction (approx. 25% below estimated energy requirements), IF generally alternates days or periods of time of complete fasting (no energy intake) or modified fasting (small amounts of energy intake) with unrestricted periods of eating or restricts normal intake to certain time periods each day. Although the benefits of these diets are often linked to weight loss induced by energy restriction, it has been suggested that IF may provide metabolic health benefits that are independent of weight loss [9] and therefore may be superior to CER in this regard. Indeed, some types of IF have been shown to improve markers of insulin sensitivity over and above that seen with CER, although this may depend on timing of consumption (i.e., early time-restricted feeding) [10] or length of fasting (i.e., consecutive fasting days versus single-day fasting) [11]. Conversely, there appears to be a general equivalence between CER and IF with respect to their impact on blood lipids [12,13]. Given the high variability in the application of IF, it is not surprising that effects may vary.

Whereas pattern of energy restriction may impact changes in cardiometabolic health, these changes can be amplified by the addition of exercise [14]. Both endurance and resistance training have been shown to improve various health outcomes with or without energy restriction and weight loss, although the impact may differ depending on the type of exercise [14]. The majority of studies that have investigated the combined effects of CER or IF with exercise have utilised endurance-type training, with the exception of those employing time-restricted feeding, in which resistance training has been more popular [15]. This is particularly true of 5:2 IF, and to date, no study has directly compared the effect of this style of IF with that of CER when combined with resistance training on cardiometabolic health parameters. Due to the variability in impact on health shown associated with both type of IF and exercise, it is important to investigate the effects of various combinations of exercise and dietary patterns.

Although exercise and energy restriction are well-known to improve cardiometabolic health, the success of any lifestyle intervention is largely dependent on an individual’s ability to comply [16], and dietary compliance, in particular over the long-term, is notoriously poor [17]. Because IF includes some periods of relief from dietary restriction, it has been proposed that it may facilitate increased compliance in some individuals [18]. However, many factors are likely to influence long-term adherence to and success of lifestyle modification, including those of a biological, behavioural, psychosocial and environmental nature [18]. For example, the degree of hunger experienced during weight loss has been shown to be a predictor of future weight regain [19], whereas mood and emotional state are also key drivers in some individuals, particularly amongst regular dieters [20]. It is important to consider how any diet targeting weight loss affects each of these variables, as it may help to determine the likelihood of compliance and therefore long-term success. This knowledge may also assist health practitioners in identifying and overcoming potential deterrents for individuals when prescribing energy-restricted diets. Unfortunately, measures of these factors are inconsistently employed in IF studies.

The purpose of this study is to present secondary outcomes from a 12-week randomised, controlled trial investigating the impact of isocaloric 5:2 IF and CER diets paired with resistance training on body composition in untrained young adults [21]. The aim of this secondary data analysis is to determine the effects of both interventions on cardiometabolic health markers, as well as the impact on self-reported dietary compliance, perceived hunger, cravings, mood and energy levels. Finally, we report on participants’ intentions to continue with their prescribed diets post intervention.

## 2. Materials and Methods

Detailed intervention methods have been previously described [21]. Briefly, 34 generally healthy (17 men and 17 women), recreationally active but untrained individuals with a BMI of 22.0–35.0 kg/m^2^ aged between 18 and 35 years were randomised to undertake 12 weeks of resistance training (3 sessions per week; two 45-minute supervised session and one 30-minute unsupervised session) and either a continuous energy restriction (CERT group) or 5:2 intermittent fasting (IFT group) diet. The aim of both diet prescriptions was to provide an average 20% energy restriction and ≥1.4 g of protein per kilogram of body weight per day. Those in the IFT group were asked to fast on non-consecutive, non-training days each week. Fasting days involved consumption of high-protein shakes and soups, as well as non-starchy vegetables, in order to maintain moderate protein intake while providing ~20% of energy requirements. Participants aimed for 100% energy requirements on the remaining 5 days. Those in the CERT group aimed to consume 80% of energy requirements daily for the entirety of the intervention. In order to assess dietary intake, participants were asked to complete a 3-day food diary using a mobile phone application during weeks 1, 6 and 12. Results of are reported in the primary outcomes study for this intervention [21]. The original study was powered to detect differences in the primary outcome (lean body mass). This study was retrospectively registered with ACTRN: ACTRN12620000920998, September 2020.

### 2.1. Cardiometabolic Health Blood Markers

Fasted blood samples were taken from each participant prior to and after the completion of the 12-week intervention in order to assess the impact of each intervention on blood markers of cardiometabolic health. Samples were taken between 7.00 a.m. and 11.00 a.m. after a minimum 8 h fast (although participants were encouraged to consume water on the night before and the morning of testing). Approximately 20 mL of blood was drawn into a serum-separating tube from a vein in the antecubital region. The tube was left to sit for at least 20 min but less than 40 min to allow for clotting. The samples were then centrifuged for 12 min at 3000 RPM to separate the serum. Serum was then aliquoted and stored at −84 degrees Celsius before being packed in dry ice and sent via courier to an external lab for analysis (Melbourne Pathology, Victoria, Australia). Serum samples were analysed for total serum cholesterol (TC), low-density lipoprotein cholesterol (LDL-C; calculated via Friedewald’s formula), high-density lipoprotein cholesterol (HDL-C), triglycerides, blood glucose, insulin and high-sensitivity C-reactive protein (hsCRP). Homeostatic model assessment of insulin resistance (HOMA-IR) was calculated using the equation ([blood glucose x insulin]/22.5) [22].

### 2.2. Self-Reported Dietary Compliance, Mood, Hunger and Satiety Levels and Post-Intervention Intentions

For each day of the 12-week intervention, participants were asked to complete an online survey. Participants were asked to complete the survey just prior to going to bed each day, consistent with previous research [23]. In order to promote completion of the survey, participants were sent as many as 5 text message reminders per week. Each participant received a unique survey link that included a total of 17 questions to assess levels of hunger, satiety, energy, mood and compliance. These questions were adapted from previously validated visual analogue scales [24,25] into 0-to-10-point Likert scales in order to facilitate use on a mobile phone. In the survey, 4 questions pertained to hunger, 4 to cravings, 4 to energy levels and 4 to mood. Additionally, there was a final question asking participants to rate, on a scale of 0 to 10, how compliant they felt they were with their diet that day, based on meeting their predefined energy and protein requirements. For analysis, these questions were combined into 5 distinct measures; hunger, cravings, energy levels, mood and compliance, which are shown in Table 1.

Scoring of each measure was undertaken by combining the responses (0–10) from the Likert scale for each of the 4 questions. For ease of interpretation, when higher scores indicated a negative response for a question category (e.g., How sleepy do you feel? for the energy levels category), values were transformed into positive scores by subtracting the recorded Likert scale score from 10. Thus, all scores were presented as positive values, and each survey category had a maximum score of 40, except for compliance (maximum score of 10). Additionally, because the original cravings questions were validated in such a way that higher scores indicated lower cravings, all questions for this section were also transformed (as per Table 1) when presenting results to ensure easier interpretation. Thus, higher scores on the cravings scale presented in the data represent higher levels of cravings.

Finally, at the end of the intervention, participants were asked to rate how easy they found the diet to comply with on a scale of 0 to 10, whether they thought they would continue with the diet after the intervention had finished and whether or not they would have preferred to be in the alternate intervention group. In an open-ended question, participants were also asked to explain what they felt was the most difficult part of the intervention.

### 2.3. Statistics

#### 2.3.1. Cardiometabolic Health Data

All results are presented as mean (±SD). Normality of variables was assessed utilising the Shapiro-Wilk test and visual inspection of Q-Q plots. Assumptions of normality were violated for hsCRP, triglycerides, insulin and HOMA-IR. Insulin and HOMA-IR were log10-transformed to achieve normality; however, normality could not be achieved for hsCRP and triglycerides. Three outlying hsCRP values (>10.0 mg/L) were removed, as these were likely to be due to acute illness. The Wilcoxon signed rank test was used to analyse differences in non-normal variables from baseline to post intervention. For normally distributed variables, linear mixed models were used to test for main effects of time, group and sex, as well as all 2- and 3-way interactions. As there was a significant difference between groups for baseline TC and a trend toward a difference in LDL-C, baseline values were centred and included as covariates in the model when analysing effects of the intervention on TC and LDL-C. Additionally, in order to account for the impact of weight loss, changes in weight from pre- to post intervention were also centred and included in the model for these variables. All model assumptions for linear mixed modelling were verified for all analyses. Individual changes are also represented graphically for each variable. Baseline data were analysed for differences between groups using independent *t*-tests and Mann–Whitney U tests for normal and non-normal data, respectively. All analyses were conducted using IBM SPSS Statistics version 25 (IBM Corp., Armonk, NY, USA). A two-tailed *p*-value of <0.05 was considered significant for all tests.

#### 2.3.2. Self-Reported Dietary Compliance, Mood, Hunger and Satiety Levels and Post-Intervention Intentions

Cronbach’s alpha was used to quantify the internal consistency of survey questions within the same measure (hunger, cravings, energy levels and mood). Alpha coefficients above 0.7 were indicated high internal consistency. Linear mixed models were used to analyse survey responses for main effects of time, group and sex, and all 2- and 3-way interactions using the mean of each week’s responses. Furthermore, the same method was used to analyse differences on fasting and non-fasting (fed) days for the IFT group, with the effect of group replaced by condition (fasted or fed). Subsequently, pairwise comparisons using Sidak’s adjustment for multiple comparisons were utilised to determine in which weeks mean values were significantly different when a main effect of time was noted. All model assumptions for linear mixed modelling were verified for all analyses. The means for weeks 1, 6 and 12 are presented, as well as continuous weekly data, which are presented graphically using mean values and 95% confidence intervals. Approximately 30% of data in this study were missing. Unfortunately, due to the small sample size, no imputation was conducted. Correlations between survey responses for each group were calculated by Spearman’s rank order correlation due to the non-normal nature of the survey data. Post-intervention questionnaire responses were analysed using descriptive analysis of responses or, for the question regarding ease of compliance, using a Mann–Whitney U test due to non-normality of the data. Similarly, compliance with the exercise routine was analysed using the Mann–Whitney U test. All analyses were conducted using IBM SPSS Statistics version 25 (IBM Corp., Armonk, NY, USA). A two-sided *p*-value of <0.05 was considered significant for all tests.

## 3. Results

### 3.1. Participants

Baseline characteristics of participants are reported in Table 2. A total of 34 participants completed the intervention (IFT = 17, CERT = 17). There were no significant differences between groups in terms of age, height, weight or BMI.

Participants completed, on average, 22.4 ± 1.76 of a possible 24 supervised resistance training sessions and 7.5 ± 2.1 of a possible 12 home workout sessions (the latter was self-reported). There was no difference between groups (supervised sessions: IFT group 22.0 ± 2.1, CERT group 22.8 ± 1.3, *p* = 0.47; unsupervised sessions: IFT group 7.8 ± 2.2, CERT group 7.1 ± 2.0, *p* = 0.24).

### 3.2. Baseline Cardiometabolic Blood Markers

Of the 34 participants, only 31 successfully had blood taken (IFT = 15, CERT = 16). Blood draws were attempted and failed on 3 participants. Baseline values for blood lipids, glucose, insulin, HOMA-IR and hsCRP for the remaining participants (*n* = 31) are presented in Table 3. Total cholesterol levels were significantly higher in the IFT group compared to the CERT group (*p* = 0.04), independent of sex. No other significant differences were noted between groups at baseline.

### 3.3. Cardiometabolic Blood Markers before and after Diet and Exercise Invervention

Average values for blood lipids, glucose, insulin, HOMA-IR and hsCRP before and after the dietary and exercise intervention are shown in Table 4. A main effect for time was found for TC (*p* ≤ 0.001), LDL-C (*p* ≤ 0.001) and HDL-C (*p* = 0.049), indicating reductions in each of these markers. There was a significant time x group interaction for TC (*p* = 0.01) and LDL-C (*p* = 0.01), with those in the IFT groups experiencing a reduction compared to those in the CERT group after adjustment for baseline levels and changes in weight. Furthermore, there was a significant time x sex x group interaction for TC (*p* = 0.04), indicating that the effect of the intervention over time varied depending on sex of the participants, with a trend towards a time x sex x group interaction for LDL-C (*p* = 0.07) (time x sex x group interactions not shown in table). There was a significant main effect of sex on HDL-C levels (*p* = 0.049), as well as a time x sex interaction (*p* = 0.001), with women demonstrating a greater reduction in HDL-C levels compared to men, irrespective of intervention group. No other main effects or interactions were found for any other variable.

**Figure 1:**
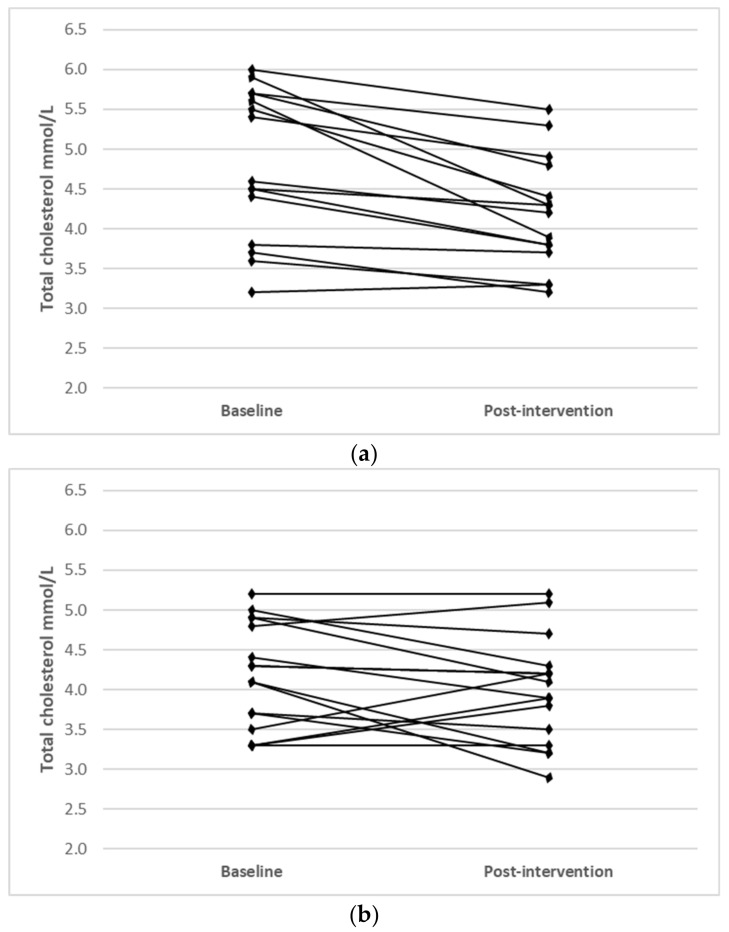

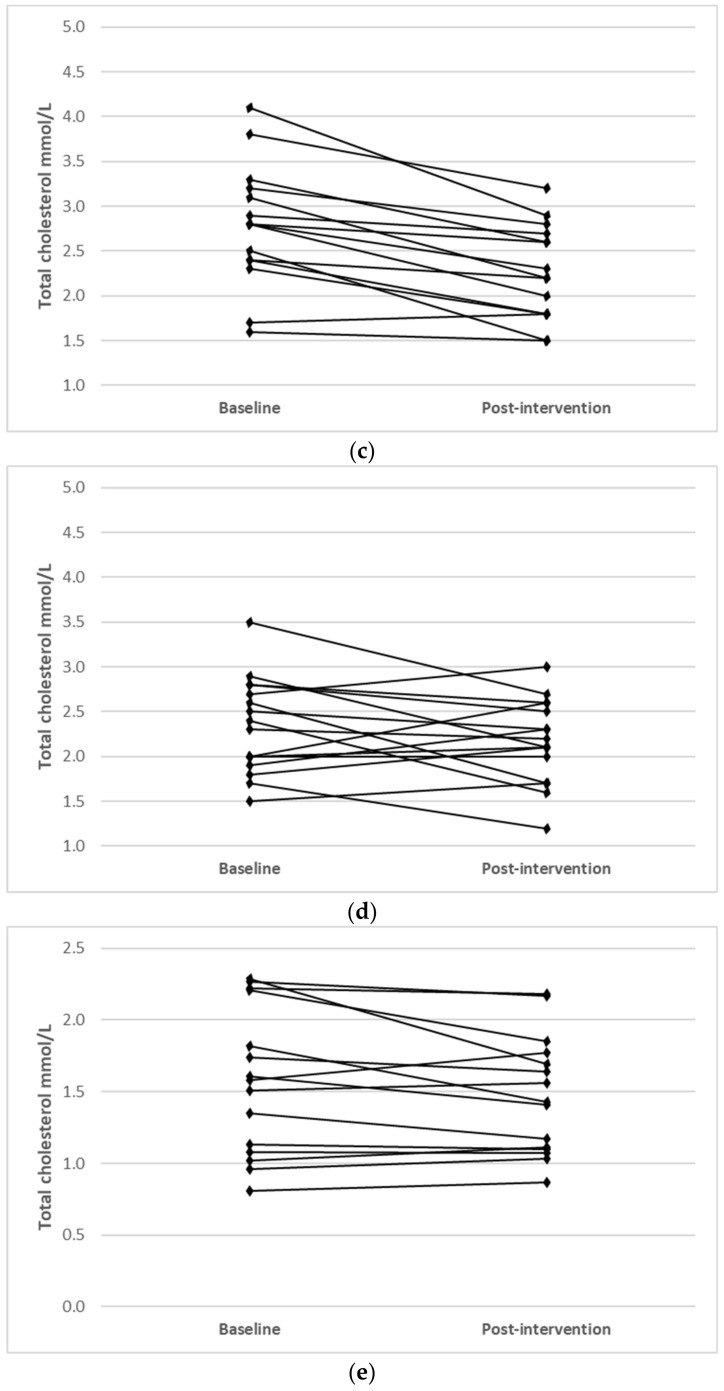

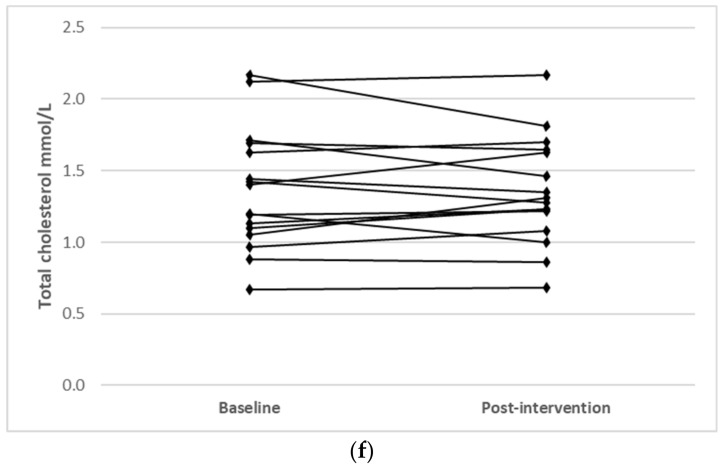
Individual changes from baseline to post intervention in (**a**) TC (IFT group), (**b**) TC (CERT group), (**c**) LDL-C (IFT group), (**d**) LDL-C (CERT group), (**e**) HDL-C (IFT group) and (**f**) HDL-C (CERT group).

Individual changes for participants where main effects were noted (TC, LDL-C and HDL-C) are shown in Figure 1.

### 3.4. Self-Reported Dietary Compliance, Hunger, Craving and Mood Levels during the Intervention Period

#### 3.4.1. Internal Consistency

Cronbach’s alpha was used to measure the level of internal consistency for survey questions grouped in the hunger, cravings, energy levels and mood scores. Based on week 1 responses, alpha values for hunger (0.902), cravings (0.728), energy levels (0.626) and mood (0.911) indicate relatively high internal consistency for most domains.

#### 3.4.2. Self-Reported Dietary Compliance, Hunger, Cravings and Mood in IFT and CERT Participants

**Figure 2:**
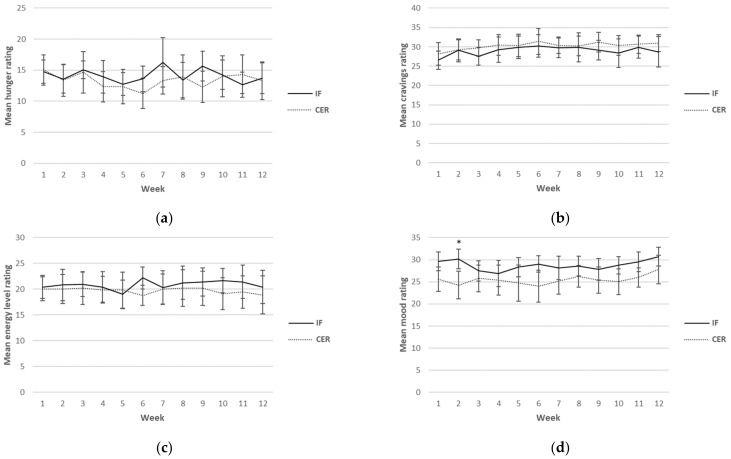

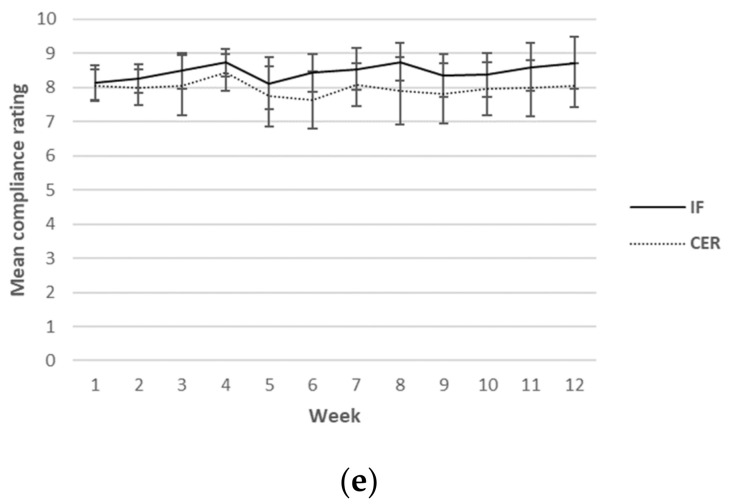
Weekly mean ratings for hunger (**a**), cravings (**b**), energy levels (**c**), mood (**d**) and compliance (**e**) over 12 weeks for CERT and IFT participants. IFT = Intermittent Fasting Diet Group, CERT = Continuous Energy Restriction Diet Group. * No overlap in 95% confidence intervals.

Self-reported ratings for hunger, cravings, energy levels, mood and compliance were collected each day over the 12-week intervention period. Although this was a continuous measure (i.e., surveys were completed daily), for ease of reading, only results from weeks 1, 6 and 12 are reported in Table 5. A graphical representation of the weekly mean values can be found in Figure 2. There was a main effect found for sex for hunger, indicating higher values in men (*p* = 0.03). Furthermore, a main effect for time for mood (*p* = 0.02) was found, indicating an increase in mood over time in both groups. No other main effects or any interactions were found.

Weekly mean reported scores over the 12 weeks for hunger, cravings, energy levels, mood and compliance are shown graphically according to diet group in Figure 2.

#### 3.4.3. Self-Reported Dietary Compliance Hunger, Cravings and Mood Levels in IFT Participants on Fasting Versus Fed Days

Self-reported ratings for hunger, cravings, energy levels, mood and compliance for the IFT group according to sex and condition (fasting days versus fed days) are presented in Table 6. There was a main effect for the conditions of hunger (*p* ≤ 0.001) and cravings (*p* ≤ 0.001), indicating lower scores for hunger and cravings on fed versus fasting days. Furthermore, there was a main effect of sex for hunger (*p* = 0.006), cravings (*p* = 0.04) and compliance (*p* = 0.02), indicating lower overall hunger, cravings and compliance ratings in women than men (including both fasting and fed days).

Correlations between each of the survey measures according to diet group are shown in Table 7. In the CERT group, there were significant positive correlations between energy levels and mood (*p* ≤ 0.001), as well as mood and compliance (*p* ≤ 0.001). There were also significant negative correlations between hunger and cravings (*p* ≤ 0.001), hunger and mood (*p* ≤ 0.001) and compliance (*p* ≤ 0.001); cravings and mood (*p* ≤ 0.05), and cravings and compliance (*p* ≤ 0.001). In the IFT group, there was a significant negative correlation between: hunger and cravings (*p* ≤ 0.001), and significant positive correlations between energy levels and mood (*p* ≤ 0.001); and mood and compliance (*p* ≤ 0.001).

Responses to the post-intervention questionnaire are presented in Table 8. There was no significant difference between how easy each group found it to comply with their allocated diet. Only four participants in the IFT group and three in the CERT group reported that they would have preferred to have been in the other dietary group. Additionally, whereas 14 participants in the IFT group and 15 in the CERT group indicated that they would continue with their diet after the intervention period, only 3 indicated that they would keep their diet the same as it had been prescribed. Common modifications that participants intended to make to their diets were lowering protein intake (*n* = 4) or, in the IFT group specifically, reducing the number of fasting days (*n* = 5) or changing intake to solid food or no intake at all on fasting days (*n* = 5). The most common difficulty noted by participants was complying with their diet during social encounters (IFT = 13, CERT = 14).

## 4. Discussion

The main findings from this secondary outcomes analysis were that both energy-restricted diets significantly reduced TC and LDL-C levels over the 12-week intervention period. These reductions were significantly greater in the IFT group compared to the CERT group, even after adjustment for baseline values and weight loss. HDL-C was also significantly decreased, albeit only in females. There were no significant differences in triglycerides, hsCRP or measures of insulin resistance over time or between interventions. Both groups reported high levels of dietary compliance and low levels of hunger. Finally, the majority of participants displayed an intention to continue with their respective diets, albeit generally in modified forms, emphasising that flexibility may be required when considering the long-term feasibility of different energy-restricted diets.

### 4.1. Cardiometabolic Biomarkers

The cohort of participants enrolled in the present study was, on average, overweight but metabolically healthy according to their baseline BMI and cardiometabolic blood markers, respectively. Over the 12-week intervention period, individuals undertaking IF displayed significantly greater reductions in TC and LDL-C compared to those undertaking CER. These results are somewhat in contrast to results of a recent systematic review and meta-analysis that found CER to be more effective at reducing TC compared to IF, although comparable effects on LDL-C levels were noted between diets [26]. In the present analysis, there was a significant time x group x sex interaction for TC and a trend toward significance for LDL-C, indicating that differences in TC and LDL-C levels between the IFT and CERT groups were likely driven by a varying response to the intervention, depending on sex. There was a notable lack of change in females undertaking CER (TC: 0.0% and LDL-C: +2.5%), whereas all other intervention/sex combinations demonstrated reductions in the order of ~10% to 20% in these markers. Although it is known that the amount of weight loss [27] and baseline values of TC and LDL-C [26] can influence changes in these markers during lifestyle interventions, this interaction remained significant after adjusting for both. It is not readily apparent why women in the IFT group experienced greater changes compared to those in the CERT group. Some previous studies in animal models have demonstrated sex-specific changes in response to calorie restriction [28,29], and it is possible that hormonal, divergent nutrient-sensing pathway activation in response to energy restriction and other mechanisms may be at play [30]. Nonetheless, in humans, research comparing CER and IF in females specifically have shown no differences in changes to TC or LDL-C (albeit utilizing endurance rather than resistance training) [31,32]. Given the limited number of studies in females within this area and the limited sample size in our study, more research is clearly needed to clarify potential sex differences.

Markers of glucose regulation were not significantly impacted over the 12-week period, regardless of dietary intervention and exercise training. However, male participants in both dietary groups demonstrated an average reduction in HOMA-IR of approximately 14–16%. Similarly, females also demonstrated an average reduction in HOMA-IR of ~22% but only within the CERT group. This is in contrast to the observations in TC and LDL-C levels discussed above, where changes were observed in females in the IFT group only. The absence of improvement in HOMA-IR among females undertaking IF was likely driven by a lack of change in insulin levels (+2%), whereas all other groups demonstrated reductions of between 14–26%, on average. Whereas this potentially points to sex-specific responses, as discussed previously, the findings are congruent with some previous human research demonstrating increases in fasting insulin in overweight females following a period of IF [33]. Hutchison and colleagues [33] observed increased fasting insulin levels when measured after a fed day (although decreased levels after a fasted day) following 8 weeks of alternate-day fasting in individuals who were prescribed a protocol designed to maintain weight. The participants in the Hutchison study, who were, on average, much older than the participants in the current analysis (~50 vs. ~24 years old) did show similar modest weight loss compared to the IFT women in our group (~2.7 kg; see previously published findings [21]), and this could explain the lack of beneficial effects on glucose regulation. Hutchison et al. [33] included another energy-restricted alternative day fasting intervention group that experienced greater amounts of weight loss (5.4 kg), who experienced significant reductions in fasting insulin when measured after both a fed and fasted day. Differences between these groups in terms of insulin levels were only significant when tested after a fed day. As our participants were only tested after a fed day, we cannot determine whether a reduction in insulin would have been observed if tested after a fasted day. Nonetheless, this raises the possibility that meaningful amounts of weight loss may be required for IF to confer consistently beneficial effects on glucose homeostasis. It is important to note that the length (i.e., consecutive fasting days) [11] or timing of fasts (i.e., early time-restricted feeding) [10,34] may be a determining factor with respect to whether or not IF provides additional benefits for glucose homeostasis. When utilizing a 5:2 IF regime that involved consecutive fasting days, Harvie et al. [31] showed a benefit over that of CER for HOMA-IR, despite similar weight loss between groups. Thus, it is possible that the protocol in the present study did not invoke fasting periods of sufficient duration or promote consumption at appropriate times to result in improvements in glucose regulation over and above that caused by weight loss.

### 4.2. Dietary Compliance, Mood, Hunger and Cravings

Participants reported similarly high rates of dietary compliance (~80%) in both the IFT and CERT groups. These results are comparable to those of most other studies that have compared 5:2 IF and CER, which reported compliance rates in the range of 56–97% over 3 months, with no differences between diets [31,35,36,37]. However, a number of factors in the present study likely promoted high levels of dietary compliance in both groups. First, participants were in regular contact with the study dietitian, who was also the strength and conditioning coach, during the supervised resistance training sessions, and frequency of contact likely improves compliance in dieting individuals [38]. Secondly, customised meal plans for each participant that were tailored to individual preferences and habitual diet [39,40], as well as the provision of meal replacement shakes [41] on fasting days for those in the IFT group. may have further helped to promote compliance. Lastly, participants were asked to self-record their dietary intake three times during the course of the study; however, many decided to track their intake continuously throughout the 12-week intervention. Self-monitoring dietary intake has been consistently shown to improve compliance with weight-loss regimens [42]. Importantly, these strategies are largely consistent with previous studies that have compared 5:2 and CER, which have generally provided regular dietary counselling or motivational support while also providing meal plans or food portion lists for individuals [31,32,36,37,43]. Although these strategies may help improve study fidelity, it is intriguing to consider how these diets might compare without the same level of support. Nonetheless, our findings add further weight to the notion that individuals show high levels of compliance to either diet in the short-to-medium term when combined with adequate professional support.

In the current study, participants reported relatively low levels of hunger. It is often proposed that hunger may be a limiting factor with respect to the success of restrictive diets, especially with IF [43]. Whereas a number of other studies have shown that hunger does not differ between those following an IF-style diet compared to CER [32,35], one longer-term intervention (1 year) showed that hunger levels were higher in those undertaking IF [43]. Together with our results, this suggests that hunger is unlikely to undermine compliance with either diet, at least in the short-to-medium term, although more longer-term studies including measures of hunger are necessary. Conversely, cravings appeared to be relatively high in both groups in the current study, which could compromise long-term compliance with either diet. Despite similar levels of hunger and cravings reported between dietary groups, there were clear differences in such ratings between fasting and fed days in the IFT group. Participants reported significantly higher levels of hunger and cravings, as well as slightly reduced mood and energy levels on fasting days compared to fed days. However, compliance was still high on fasting days, potentially indicating the acceptability of short-term, severe restriction.

Mood was positively correlated with compliance in both groups, perhaps signifying greater ability of participants to comply with their assigned diet when their mood was better (although not precluding the possibility that greater compliance led to improved mood). The CERT group showed a significant negative correlation between hunger and mood, as well as between hunger and compliance—associations not observed in the IFT group. On the other hand, participants in the IFT group appeared to experience a reprieve from high levels of hunger and poorer mood on non-fasting days, reporting significantly lower ratings compared to fed days. This is an important consideration for practitioners when recommending dietary interventions, given that an individual’s capacity to self-regulate may be limited. Furthermore, given the tendency of individuals to adopt an ‘all-or-nothing’ approach to dieting, a dietary lapse can often lead to reactionary overeating [44], compromising weight loss. CER provides a greater opportunity for this to occur than IF, which could be exacerbated if this type of dieting also causes poorer mood. Nonetheless, this is speculative, and there remains a paucity of long-term studies that include measures of compliance, hunger and mood.

Following the completion of the intervention period, participants were asked to rate the ease with which they were able to comply with their diet. Participants rated IFT and CERT as a 6.6 and 7.1 out of 10, respectively. These values are lower than those reported in other studies at 3, 6 and 12 months when comparing 5:2 IF and CER [43]. This may have been due to the nature of the prescribed diets, namely a higher recommended protein intake and liquid meals on fasting days for the IFT group. Nearly half of all participants in the current study reported difficulty meeting the protein targets (IFT = 5, CERT = 10), and half of those in the IFT group noted that they would have preferred solid foods instead of liquid meals on fasting days. This was reflected further in the intentions of the participants at the end the intervention period. Whereas a large majority indicated they would continue with their diet protocols post intervention, only a few intended to continue as originally prescribed. Common changes that participants planned to make were a reduction in protein, regardless of dietary group, as well as a reduction in the number of fasting days and/or the use of solid meals instead of liquid meals on fasting days for those in the IFT group. Interestingly, in one of the few other studies to utilise liquid meals for their 5:2 IF fasting group, Harvie et al. [31] showed that a much smaller proportion of 5:2 IF participants (58%) planned to continue with their diet after the 6-month intervention compared to the CER group (85%). Conversely, Sundfør et al. [4] showed that participants in both 5:2 IF (utilising consumption of solid food on fasting days) and CER diet groups had comparably strong intentions to continue with their diet after 12 months. Thus, although both diets appear to be tolerable, flexibility and customisation with regard to how energy deficits are implemented may be important for long-term compliance. It should also be noted that the vast majority of participants from both groups (CERT = 14, IFT = 13) reported difficulty complying with their diet during social situations, with 1 participant in the IFT group also reporting avoiding going out on fasting days. This could also have implications for long-term compliance and quality of life, further emphasising the need for interventions that allow for some flexibility.

There were a number of strengths of this study. First, both diets were matched for protein intake and energy restriction; therefore, any differences observed between groups may be more likely due to differing intake patterns (i.e., IF versus CER) rather than major differences in nutrient intake. Secondly, we utilised continuous data to analyse dietary compliance, as well as changes in hunger, cravings and mood, with questionnaires based on previously validated scales. However, our study is also subject to several limitations. Despite the continuous nature of the questionnaires, the frequency of the collection (i.e., daily) also led to decreased response rates over time and potentially introduced habituality to the nature of responses. Furthermore, a lack of preintervention testing precluded analysis of baseline differences in hunger, cravings and mood. We also failed to take into account menstrual cycles in women, which may impact a number of the variables we measured, in particular, hunger, cravings and mood. Finally and importantly, this study was powered to detect differences in the primary outcome for this intervention (lean body mass); therefore the results should be interpreted with caution and considered exploratory.

The results from our secondary outcome analysis demonstrate that IF and CER combined with resistance exercise can improve cardiometabolic risk biomarkers in overweight but metabolically healthy adults, with greater improvements in IF independent of weight change and baseline TC and LDL-C levels. Both energy restriction strategies resulted in low levels of hunger over the 12 weeks, which may have contributed to the high levels of compliance, along with the support and guidance provided in the study. Importantly, both dietary strategies were viewed favourably, with participants indicating that they would continue with their prescribed diets. However, most planned to do so with minor modifications, indicating that flexibility and customisation may be important for long-term compliance.

## Figures and Tables

**Table 1:** Questions included within each survey category for hunger, cravings, mood and energy levels.

Questions Included within Each Survey Category	
Hunger (maximum score possible 40)	1. How hungry do you feel? (+ve) 2. How satisfied do you feel? (−ve) 3. How full do you feel? (−ve) 4. How much do you think you can eat? (+ve)
Cravings (maximum score possible 40)	1. Would you like something sweet? (+ve) 2. Would you like something salty? (+ve) 3. Would you like something savoury? (+ve) 4. Would you like something fatty? (+ve)
Energy levels (maximum possible score 40)	1. How alert do you feel? (+ve) 2. How much of an effort is it to do anything? (−ve) 3. How weary do you feel? (−ve) 4. How sleepy do you feel? (−ve)
Mood (maximum score possible 40)	1. How sad do you feel? (−ve) 2. How tense do you feel? (−ve) 3. How happy do you feel? (+ve) 4. How calm do you feel? (+ve)
Compliance (maximum score possible 10)	1. How compliant do you feel you were with your diet today? (+ve)

+ve: values are positively associated with the domain; −ve: values are negatively associated with the domain and were transformed for subsequent analysis.

**Table 2:** Participant baseline characteristics.

Baseline Variable	IFT (*n* = 17; 9 Males, 8 Females)	CERT (*n* = 17; 8 Males, 9 Females)	*p*-Value ^a^
Age (years)	24.7 (4.8)	23.2 (3.9)	0.31
Height (m)	1.72 (0.1)	1.71 (0.1)	0.82
Weight (kg)	80.1 (13.8)	79.6 (13.5)	0.92
BMI (kg/m^2^)	27.0 (2.7)	27.1 (2.9)	0.93

Note: Mean (*SD*). ^a^ *p*-values reported are for independent *t*-tests between intervention groups. BMI = body mass index.

**Table 3:** Baseline values of cardiometabolic blood markers according to intervention group and sex.

Baseline Variable	IFT Males (*n* = 8)	CERT Males (*n* = 8)	IFT Females (*n* = 7)	CERT Females (*n* = 8)	IFT Group (*n* = 15)	CERT Group (*n* = 16)	*p*-Value (Group)
hsCRP (mg/L)	0.94 (0.64)	1.41 (1.63)	2.00 (1.82)	2.43 (1.72)	1.39 (1.34)	1.92 (1.69)	0.26 ^2^
TC (mmol/L)	4.49 (0.79)	4.18 (0.60)	5.17 (1.02)	4.18 (0.75)	4.81 (0.94)	4.18 (0.66)	0.04 ^1^
Triglycerides (mmol/L)	0.94 (0.26)	1.20 (0.51)	1.01 (0.36)	0.90 (0.36)	0.97 (0.30)	1.05 (0.45)	0.86 ^2^
HDL-C (mmol/L)	1.35 (0.47)	1.15 (0.32)	1.83 (0.46)	1.57 (0.43)	1.57 (0.51)	1.36 (0.42)	0.22
LDL-C (mmol/L)	2.71 (0.63)	2.49 (0.60)	2.86 (0.78)	2.19 (0.45)	2.78 (0.68)	2.34 (0.53)	0.05
Glucose (mmol/L)	4.88 (0.33)	4.90 (0.35)	4.84 (0.42)	4.76 (0.52)	4.86 (0.36)	4.83 (0.44)	0.84
Insulin (mU/L) ^3^	8.55 (4.54)	10.26 (6.22)	10.40 (2.73)	11.89 (7.69)	9.41 (3.80)	11.08 (6.81)	0.59
HOMA-IR ^3^	1.88 (1.07)	2.29 (1.60)	2.27 (0.75)	2.61 (2.02)	2.06 (0.92)	2.45 (1.77)	0.64

Note: Mean (SD). ^1^ Significantly different between IFT and CERT. ^2^ Data from Mann–Whitney U test. ^3^ Presented values are non-transformed, and all analyses were conducted on Log10-transformed values to achieve normality. HDL-C = high-density lipoprotein cholesterol, hsCRP = high-sensitivity C-reactive protein, HOMA-IR = homeostatic model assessment of insulin resistance, LDL-C = low-density lipoprotein cholesterol, TC = total serum cholesterol.

**Table 4:** The effects of 12 weeks of IFT or CERT with resistance exercise on cardiometabolic blood markers.

Diet variable	Group	Baseline	Post Intervention	Δ	Δ (%)	*P* (Group)	*P* (Time)	*P* (I)	*P* (S)
TC (mmol/L) ^5^	IFT group (*n* = 15)	4.81 (0.94)	4.18 (0.71)	−0.63	−13.1	0.20	<0.001 ^1^	0.01 ^2^	0.92
	CERT group (*n* = 16)	4.18 (0.66)	3.98 (0.67)	−0.20	4.8				
	IFT males (*n* = 8)	4.49 (0.79)	4.03 (0.68)	−0.46	−10.2				
	CERT males (*n* = 7)	4.18 (0.60)	3.79 (0.44)	−0.39	−9.3				
	IFT females (*n* = 8)	5.17 (1.02)	4.36 (0.76)	−0.81	−15.7				
	CERT females (*n* = 8)	4.18 (0.75)	4.18 (0.82)	0.00	0.0				
	IFT group (*n* = 15)	2.78 (0.68)	2.26 (0.53)	−0.52	−18.7	0.19	<0.001 ^1^	0.01 ^2^	0.20
LDL-C (mmol/L) ^5^	CERT group (*n* = 16)	2.34 (0.53)	2.17 (0.46)	−0.17	−7.3				
	IFT males (*n* = 8)	2.71 (0.63)	2.23 (0.51)	−0.48	−17.7				
	CERT males (*n* = 7)	2.49 (0.60)	2.10 (0.48)	−0.39	−15.7				
	IFT females (*n* = 8)	2.86 (0.78)	2.30 (0.58)	−0.56	−19.6				
	CERT females (*n* = 8)	2.19 (0.45)	2.24 (0.47)	0.05	2.3				
	IFT group (*n* = 15)	1.57 (0.51)	1.47 (0.41)	−0.10	−6.4	0.23	0.049 ^1^	0.08	0.001 ^3^
HDL-C (mmol/L)	CERT group (*n* = 16)	1.36 (0.42)	1.35 (0.38)	−0.01	−0.7				
	IFT males (*n* = 8)	1.35 (0.47)	1.38 (0.44)	0.03	2.2				
	CERT males (*n* = 7)	1.15 (0.32)	1.21 (0.35)	0.06	5.2				
	IFT females (*n* = 8)	1.83 (0.46)	1.58 (0.38)	−0.25	−13.7				
	CERT females (*n* = 8)	1.57 (0.43)	1.50 (0.37)	−0.07	−4.6				
	IFT group (*n* = 15)	4.86 (0.36)	4.94 (0.30)	0.08	1.6	0.91	0.09	0.55	0.35
Glucose (mmol/L)	CERT group (*n* = 16)	4.83 (0.44)	4.99 (0.34)	0.16	3.3				
	IFT males (*n* = 8)	4.88 (0.33)	4.95 (0.28)	0.07	1.4				
	CERT males (*n* = 7)	4.90 (0.35)	4.94 (0.27)	0.04	0.8				
	IFT females (*n* = 8)	4.84 (0.42)	4.93 (0.32)	0.09	1.9				
	CERT females (*n* = 8)	4.76 (0.52)	5.05 (0.41)	0.29	6.1				
	IFT group (*n* = 15)	9.41 (3.80)	8.89 (2.76)	−0.52	−5.5	0.95	0.21	0.29	0.79
Insulin (mU/L) ^4^	CERT group (*n* = 16)	11.08 (6.81)	8.71 (4.94)	−2.37	−21.4				
	IFT males (*n* = 8)	8.55 (4.54)	7.38 (2.13)	−1.17	−13.7				
	CERT males (*n* = 7)	10.26 (6.22)	8.56 (5.59)	−1.70	−16.6				
	IFT females (*n* = 8)	10.40 (2.73)	10.61 (2.43)	0.21	2.0				
	CERT females (*n* = 8)	11.89 (7.69)	8.85 (4.59)	−3.04	−25.6				
	IFT group (*n* = 15)	2.06 (0.92)	1.95 (0.60)	−0.11	−5.3	0.96	0.36	0.36	0.73
HOMA-IR ^4^	CERT group (*n* = 16)	2.45 (1.77)	1.99 (1.30)	−0.46	−18.8				
	IFT males (*n* = 8)	1.88 (1.07)	1.62 (0.49)	−0.26	−13.8				
	CERT males (*n* = 7)	2.29 (1.60)	1.93 (1.38)	−0.36	−15.7				
	IFT females (*n* = 8)	2.27 (0.75)	2.32 (0.51)	0.05	2.2				
	CERT females (*n* = 8)	2.61 (2.02)	2.05 (1.31)	−0.56	−21.5				
Non-normal variables	Group	Baseline	Post Intervention	Δ	Δ (%)	Signed rank Wilcoxon test (paired samples)			
						Group/sex		Diet group	
hsCRP (mg/L)	IFT group (*n* = 15)	1.39 (1.34)	1.39 (1.66)	0.00	0.0	Z = −1.13, *p* = 0.26		Z = 0.00, *p* = 1.00	
	CERT group (*n* = 17)	1.92 (1.69))	1.21 (1.07)	−0.71	−37.0	Z = −1.16, *p* = 0.25		Z = −1.73, *p* = 0.08	
	IFT males (*n* = 8)	0.94 (0.64)	0.59 (0.35)	−0.35	−37.2	Z = 0.94, *p* = 0.35			
	CERT males (*n* = 7)	1.41 (1.63)	1.07 (1.26)	−0.34	−24.1	Z = −1.36, *p* = 0.17			
	IFT Females (*n* = 6)	2.00 (1.82)	2.45 (2.14)	0.45	25.6				
	CERT females (*n* = 7)	2.43 (1.72)	1.36 (0.91)	−1.07	−44.0				
Triglycerides (mmol/L)	IFT group (*n* = 17)	0.97 (0.30)	0.98 (0.38)	0.01	1.0	Z = −0.53, *p* = 0.60		Z = −0.36, *p* = 0.71	
	CERT group (*n* = 17)	1.05 (0.45)	1.02 (0.41)	−0.03	−2.9	Z = −1.02, *p* = 0.31		Z = −0.35, *p* = 0.73	
	IFT males (*n* = 8)	0.94 (0.26)	0.93 (0.35)	−0.01	−1.1	Z = −0.18, *p* = 0.85			
	CERT males (*n* = 7)	1.20 (0.51)	1.06 (0.47)	−0.14	−11.7	Z = −0.84, *p* = 0.40			
	IFT females (*n* = 8)	1.01 (0.36)	1.04 (0.44)	0.03	3.0				
	CERT females (*n* = 8)	0.90 (0.36)	0.98 (0.37)	0.08	8.9				

Note: Mean (*SD*). ^1^ Significantly different than baseline at week 12 in all groups combined. ^2^ Significant time x diet group interaction. ^3^ Significant time x sex interaction. ^4^ Presented values are non-transformed, and all analysis were conducted on Log10-transformed values to achieve normality. ^5^ Analysis for TC and LDL-C adjusted for baseline values and weight change. Group = main effect for diet group, I = time x group interaction, S = time x sex interaction, Time = main effect for time, HDL-C = high-density lipoprotein cholesterol, hsCRP = high-sensitivity C-reactive protein, HOMA-IR = homeostatic model assessment of insulin resistance, LDL-C = low-density lipoprotein cholesterol, TC = total serum cholesterol, IFT = Intermittent Fasting Diet Group, CERT = Continuous Energy Restriction Diet Group.

**Table 5:** The effects of 12 weeks of IFT and CERT with resistance exercise on mean ratings of hunger, cravings, energy levels, mood and compliance.

Survey Variable	Group	Week 1	Week 6	Week 12	Δ	Δ (%)	*P* (Group)	*P* (Time)	*P* (I)	*P* (S)
Hunger (max. 40)	IFT group (*n* = 17)	14.7 (3.9)	13.6 (4.4)	13.7 (5.2)	−1.0	−6.8	0.63	0.22	0.49	0.03 ^2^
	CERT group (*n* = 17)	15.0 (5.2)	11.3 (5.1)	13.3 (6.4)	−1.7	−11.3				
	IFT males (*n* = 9)	16.5 (3.3)	15.2 (4.9)	13.9 (5.4)	−2.6	−15.8				
	CERT males (*n* = 8)	15.2 (4.6)	11.4 (3.3)	14.1 (4.7)	−1.1	−7.2				
	IFT females (*n* = 8)	12.8 (3.8)	11.8 (3.1)	13.6 (5.4)	2.1	6.3				
	CERT females (*n* = 9)	14.9 (5.8)	11.2 (6.5)	12.6 (7.8)	−2.3	−15.4				
Cravings (max. 40)	IFT group (*n* = 17)	26.5 (5.0)	30.2 (6.1)	28.7 (8.2)	2.2	8.3				
	CERT group (*n* = 17)	28.1 (6.1)	31.4 (7.0)	31.0 (4.6)	2.9	10.3				
	IFT males (*n* = 9)	25.2 (4.9)	29.2 (7.3)	27.9 (5.2)	−2.7	−10.7	0.46	0.08	0.96	0.20
	CERT males (*n* = 8)	29.3 (6.1)	30.9 (8.4)	30.6 (5.0)	1.3	4.4				
	IFT females (*n* = 8)	28.0 (5.0)	31.4 (4.7)	29.5 (10.8)	1.5	5.4				
	CERT females (*n* = 9)	27.1 (6.4)	31.9 (6.0)	31.3 (4.6)	4.2	15.52				
Energy levels (max. 40)	IFT group (*n* = 17)	20.4 (4.6)	22.2 (4.5)	20.4 (6.8)	0.0	0.0				
	CERT group (*n* = 17)	20.1 (4.9)	18.8 (4.1)	18.9 (7.7)	−1.2	−6.0				
	IFT males (*n* = 9)	21.1 (4.0)	23.2 (3.7)	20.3 (5.5)	−0.8	−3.8	0.61	0.77	0.37	0.18
	CERT males (*n* = 8)	21.5 (5.2)	20.2 (5.2)	21.7 (9.0)	0.2	0.9				
	IFT females (*n* = 8)	19.7 (5.3)	21.0 (5.3)	20.6 (8.3)	0.9	4.6				
	CERT females (*n* = 9)	18.9 (4.7)	17.6 (2.3)	16.4 (5.8)	−2.5	−13.2				
Mood (max. 40)	IFT group (*n* = 17)	29.6 (4.5)	29.0 (3.8)	30.7 (4.5)	1.1	3.7	0.06	0.02 ^1^	0.14	0.07
	CERT group (*n* = 17)	25.6 (5.8)	24.0 (7.6)	27.8 (6.8)	2.2	8.6				
	IFT males (*n* = 9)	30.0 (5.7)	29.8 (3.6)	31.1 (4.8)	1.1	3.7				
	CERT males (*n* = 8)	27.6 (3.0)	24.0 (7.4)	30.7 (2.8)	3.1	11.2				
	IFT females (*n* = 8)	29.2 (3.0)	28.2 (4.2)	30.2 (4.5)	1.0	3.4				
	CERT females (*n* = 9)	23.8 (7.1)	24.1 (8.1)	25.3 (8.5)	1.5	6.3				
Compliance (max. 10)	IFT group (*n* = 17)	8.1 (1.1)	8.4 (1.2)	8.7 (1.6)	0.6	7.4				
	CERT group (*n* = 17)	8.1 (1.0)	7.6 (1.8)	8.1 (1.3)	0.0	0.0				
	IFT males (*n* = 9)	8.3 (0.8)	8.8 (1.4)	9.4 (0.8)	1.1	13.3	0.25	0.31	0.90	0.08
	CERT males (*n* = 8)	8.5 (0.8)	7.8 (1.6)	8.3 (1.6)	−0.2	−2.4				
	IFT females (*n* = 8)	8.0 (1.2)	8.1 (0.8)	8.0 (2.0)	0.0	0.0				
	CERT females (*n* = 9)	7.7 (1.0)	7.5 (2.0)	7.8 (1.1)	0.1	1.3				

Note: Mean (*SD*). Δ represents change from week 1 to week 12. ^1^ Significantly different from weeks 4 and 9 in week 12 in all groups combined. ^2^ Significant main effect for sex. I = time x group interaction, S = main effect for sex, Time = main effect for time.

**Table 6:** Mean ratings for hunger, cravings, energy levels, mood and compliance separated by fasting and fed days in IFT participants over 12 weeks.

Survey Variable	Group	Week 1	Week 6	Week 12	Δ	Δ (%)	*P* (C)	*P* (Time)	*P* (CS)	*P* (S)
Hunger (max. 40)	IFT Males (*n* = 9) fed	11.2 (3.2)	9.7 (3.6)	9.2 (3.5)	−2.0	−17.9	<0.001 ^1^	0.32	0.34	0.01 ^2^
	IFT Males (*n* = 9) fasted	26.5 (5.2)	27.0 (6.3)	26.7 (7.6)	0.2	0.8				
	IFT Females (*n* = 8) fed	7.7 (3.1)	6.9 (4.0)	7.6 (2.7)	−0.1	−1.3				
	IFT Females (*n* = 9) fasted	23.6 (6.0)	21.8 (6.4)	22.4 (5.3)	−0.8	−3.4				
Cravings (max 40.)	IFT Males (*n* = 9) fed	11.9 (4.6)	7.0 (4.9)	8.2 (4.8)	−3.7	−31.1	<0.001 ^1^	0.21	0.94	0.04 ^2^
	IFT Males (*n* = 9) fasted	19.1 (5.4)	18.6 (5.3)	21.8 (6.7)	2.7	14.1				
	IFT Females (*n* = 8) fed	7.8 (4.7)	4.6 (3.1)	4.5 (3.6)	−3.3	−42.3				
	IFT Females (*n* = 9) fasted	19.9 (7.1)	17.4 (8.7)	17.6 (10.6)	−2.3	−11.6				
Energy levels (max. 40)	IFT Males (*n* = 9) fed	21.8 (4.7)	23.2 (4.6)	21.8 (4.8)	0.0	0.0	0.31	0.27	0.66	0.69
	IFT Males (*n* = 9) fasted	19.3 (5.4)	22.2 (5.0)	19.4 (7.4)	0.1	0.5				
	IFT Females (*n* = 8) fed	20.9 (6.7)	21.6 (5.5)	20.8 (7.2)	−0.1	−0.5				
	IFT Females (*n* = 9) fasted	17.8 (3.7)	19.6 (5.8)	20.4 (7.9)	2.6	14.6				
Mood (max. 40)	IFT Males (*n* = 9) fed	30.7 (4.7)	30.4 (3.7)	30.0 (3.8)	−0.7	−2.3	0.06	0.09	0.59	0.06
	IFT Males (*n* = 9) fasted	28.5 (6.7)	29.1 (4.2)	28.0 (4.8)	−0.5	−1.8				
	IFT Females (*n* = 8) fed	30.0 (2.8)	29.1 (3.2)	28.4 (3.8)	−1.6	−5.3				
	IFT Females (*n* = 9) fasted	26.4 (5.9)	26.5 (4.4)	27.4 (5.7)	1.0	3.8				
Compliance (max. 10)	IFT Males (*n* = 9) fed	8.1 (0.7)	8.6 (0.9)	8.7 (0.9)	0.6	7.4	0.06	0.26	0.68	0.02 ^2^
	IFT Males (*n* = 9) fasted	9.1 (1.1)	9.1 (1.2)	9.5 (0.6)	0.4	4.4				
	IFT Females (*n* = 8) fed	7.9 (1.4)	7.9 (0.6)	8.0 (1.2)	0.1	1.3				
	IFT Females (*n* = 9) fasted	8.6 (1.2)	8.4 (1.9)	8.3 (2.3)	−0.3	−3.5				

Note: Mean (*SD*). ^1^ Significant main effect for condition (fasted/fed). ^2^ Significant main effect for sex. C = main effect for condition (fasted/fed), CS = condition x sex interaction, S = main effect for sex, Time = main effect for time.

**Table 7:** Bivariate correlations between survey measures according to intervention group.

Survey Variable	Hunger	Cravings	Energy Levels	Mood	Compliance
CERT					
Hunger	1.00	−0.52 ^1^	0.04	−0.39 ^1^	−0.26 ^1^
Cravings		1.00	−0.07	−0.15 ^2^	−0.33 ^1^
Energy Levels			1.00	0.51 ^1^	0.11
Mood				1.00	0.34 ^1^
Compliance					1.00
IFT					
Hunger	1.00	−0.71 ^1^	0.10	−0.12	−0.05
Cravings		1.00	0.02	0.10	−0.02
Energy Levels			1.00	0.54 ^1^	0.06
Mood				1.00	0.26 ^1^
Compliance					1.00

Note: Spearman’s rho. ^1^ Significant correlation (*p* < 0.001). ^2^ Significant correlation (*p* < 0.05)

**Table 8:** Post-intervention questionnaire responses to the dietary intervention in IFT and CERT participants.

How Easy Was It to Comply with Your Diet? (0 = Not Easy at All 10 = Extremely Easy)	Mean Score (SD)	*p*-Value
IFT	6.6 (1.8)	0.36
CERT	7.1 (1.3)	
Would you have preferred to have been in the other diet group?	Yes	No
IFT	4	13
CERT	3	14
Will you continue with your diet after the intervention period has finished?	Yes	No
IFT	14 *	3
CERT	15 *	2

* Only one participant in the IFT group and two in the CERT group indicated that they would continue with their diet as prescribed; the remainder from each group noted that they would modify it in some way.

## Data Availability

The data presented in this study are available on request from the corresponding author. The data are not publicly available due to ethical considerations.
